# Impairment of attention network function in posterior circulation ischemia-evidence from the Attention Network Test

**DOI:** 10.3389/fnhum.2022.1001500

**Published:** 2023-01-06

**Authors:** Na Li, Chuanjin Li, Xiaohui Xie, Gang Liu, Kai Wang, Wendong Zhang, Jin Fan

**Affiliations:** ^1^The Third Department of Encephalopathy, The Second Affiliated Hospital of Anhui University of Chinese Medicine, Hefei, Anhui, China; ^2^The First Affiliated Hospital of Anhui University of Chinese Medicine, Hefei, China; ^3^Department of Neurology, The First Affiliated Hospital of Anhui Medical University, Hefei, Anhui, China; ^4^Department of Encephalopathy, The Second Affiliated Hospital of Anhui University of Chinese Medicine, Hefei, China; ^5^Department of Psychiatry and Neuroscience, Mount Sinai School of Medicine, New York, NY, United States

**Keywords:** posterior circulation ischemic, Attention Network Test, cognition, alerting, orienting, executive control

## Abstract

**Objective:**

This study aimed to investigate the effect of posterior circulation ischemia (PCI) on attention network function and to determine whether PCI is holistic or selective attention network deficit and which attention network is affected.

**Methods:**

Thirty-six PCI patients aged 30 to 75 were assessed using the Attention Network Test and the Mini-Mental State Examination (MMSE). There were no significant differences in age, sex, and education between PCI group and the control group (*n* = 32). All data were statistically analyzed by SPSS 23.0 software.

**Result:**

There were no significant difference in the MMSE scores between the two groups. Compared with the control group, the PCI group had significantly shorter response time for alerting and orienting network. The executive control network response time was significantly longer in PCI group than in the control group. The overall mean response time was also significantly longer in PCI group than in normal control group. There was no significant difference in mean accuracy between the two groups.

**Conclusion:**

The alerting, orienting, and executive control networks were significantly less efficient in PCI group than in the control group (*P* < 0.01). This indicates impaired attention network in PCI patients. Since transient nerve seizures caused by vertebrobasilar ischemia may precede posterior circulation stroke, early assessment of cognitive function in patients with PCI is particularly important, and ANT is an excellent tool for this assessment.

## Highlights

– Posterior circulation ischemia (PCI) can lead to attention deficits.
– Attention Network Test (ANT) can be used to assess cognitive deficits.
– Posterior circulation ischemia patients have an overall longer response time.
– Posterior circulation ischemia impairs alerting, orienting, and executive control attention networks.
– Attention Network Test can be used to assess network deficits in PCI patients.

## Introduction

Ischemic cerebrovascular diseases accounts for 80% of cerebrovascular diseases worldwide (Kyu et al., 2018). Posterior circulation infarction (POCI) accounts for 20% of ischemic strokes (Amin-Hanjani et al., 2019), which leaves a large number of post-stroke survivors and increases the burden on society. Although the major motor and sensory pathways are usually intact after POCI, vertigo becomes its prodrome (Atzema et al., 2016; Newman-Toker, 2016), which may help with the early diagnosis of stroke. Similarly, in posterior circulation ischemia (PCI), the primary symptom is vertigo, and the blood supply mainly includes cerebellum, brainstem, occipital lobe and ventral thalamus, etc. (Searls et al., 2012). The main manifestations are nausea and vomiting, diplopia, numbness or weakness of limbs, arrhythmia or dysphagia. The most common causes of PCI are atherosclerotic lesions, embolism, and small penetrating artery disease, which lead to functional defects in the inner ear, brainstem, and cerebellum (Vuilleumier et al., 1995; Caplan, 2000; Caplan et al., 2004; Savitz and Caplan, 2005). Clinical evidence shows that posterior circulation ischemic vertigo (PCIV) is at high risk of recurrence, with anxiety, depression, and other psychiatric disorders (Gulli et al., 2009; Herdman et al., 2020), which should cause alarm among clinicians. Most PCIV patients are middle-aged and elderly. They often have recurring seizures and are unable to fully recover, which hinders their quality of life (Dong et al., 2020). The effect of PCI on cognitive function has gradually attracted attention, and studies have found that PCI is a risk factor for cognitive impairment (Ito et al., 2010; Kocer, 2015; Tu et al., 2018; Yaghi et al., 2020).

**FIGURE 1:**
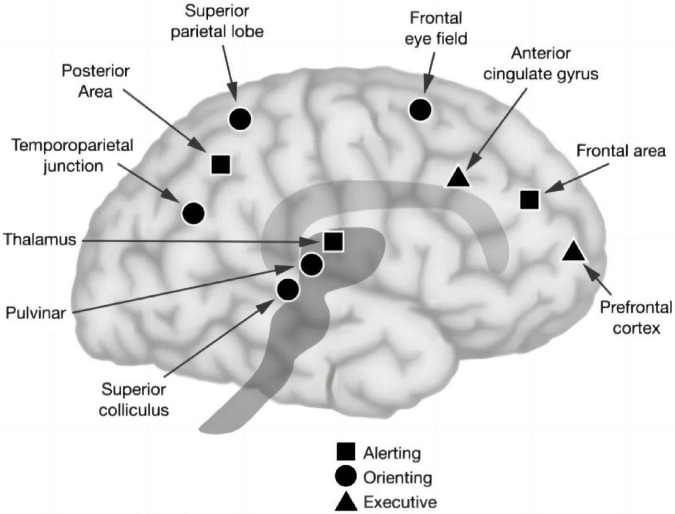
Anatomy of three attentional networks: alerting, orienting, and executive control attention (Posner and Rothbart, 2007).

Attention is the cornerstone of cognition, which refers to the direction and concentration of mental activities on an object or event. It is commonly accompanied by other psychological processes, such as perception, thinking, memory, and imagination, and is an essential attribute for the generation and implementation of all psychological processes. Attention plays an important role in cognitive psychology and cognitive neuroscience (Posner and Rothbart, 2007). In recent years, more and more scholars have paid attention to the attention of patients with neurodegenerative diseases. Some studies have confirmed that vertigo patients have difficulty in concentration (Wuehr et al., 2017; Kane et al., 2019). Vertigo is usually the first symptom of posterior circulation ischemia. Therefore, we hypothesize that posterior circulation ischemia could cause cerebral ischemia and hypoxia, which would lead to difficulty in concentration. Studies such as those by Posner et al (Fan et al., 2002; Petersen and Posner, 2012). proposed that attention is mediated by several neural infrastructures, which can be divided into three subsystems: alerting, orienting, and executive control. The alerting network activates an individual’s state of stress to maintain tension and produce staged responses to alarm signals. It is located in the thalamus, frontal, and parietal regions of the right hemisphere and utilizes the norepinephrine system, whose function is critical for optimizing performance (Fan et al., 2003). The orienting network involves an individual’s ability to selectively focus on one or more projects/tasks. Previous studies have shown that this network consists of temporoparietal connectivity and the superior parietal lobe, inferior parietal lobe, frontal field, thalamo-occipital nucleus, and reticular nucleus, and is regulated by the cholinergic system (Nobre, 2001; Corbetta and Shulman, 2002). Finally, the executive control network underlies the ability to monitor and resolve conflicts in the presence of competing information, which relies on the prefrontal and anterior cingulate cortices, as well as on subcortical structures, such as the basal ganglia and cerebellum, and is regulated by the dopaminergic system (Moriya, 2018; Figure 1).

Fan et al. (2003) designed the Attention Network Test (ANT) software based on the alerting, orienting, and executive control network theory of Posner and Petersen’s (1990). Fan et al. (2002) has shown that the ANT clearly involves all three attention networks and can be used to measure the efficiency of every network, as simple as taking data from children, patients, and animals. The ANT, which can be used to assess functional independence, is reliable in estimating alerting, orienting, and executive control functions for a single subject and further indicates that the efficiency of the three networks is unrelated. The ANT can not only perform a fine quantitative analysis of the subjects’ attention network function but also serve as a basis for neuropsychological evaluation and has auxiliary diagnostic value (Xia et al., 2022). This test can be useful in exploring whether patients with PCI have generalized attention deficit or selective attention network disorder. The software has been widely applied in studies of depression, epilepsy, schizophrenia, Alzheimer’s disease, and insomnia in the general population (Perrier et al., 2015; Wang et al., 2016, 2020; McDonough et al., 2019; Arkin et al., 2020; Sarrias-Arrabal et al., 2020; Wang and Guo, 2020). However, there have been no reports investigating the association between PCI and the three attention networks.

In this study, we aimed to assess attention network functioning in older adults with and without PCI and to determine whether attention networks are generally impaired in patients with PCI. Patients who were newly diagnosed with PCI and who had not yet been treated with drugs to ameliorate the condition were asked to undergo the ANT. To our knowledge, this is the first study to explore the effects of PCI on the cognitive attention network, which will expand the scope of research in the treatment of patients with PCI.

## Materials and methods

### Study design

**FIGURE 2:**
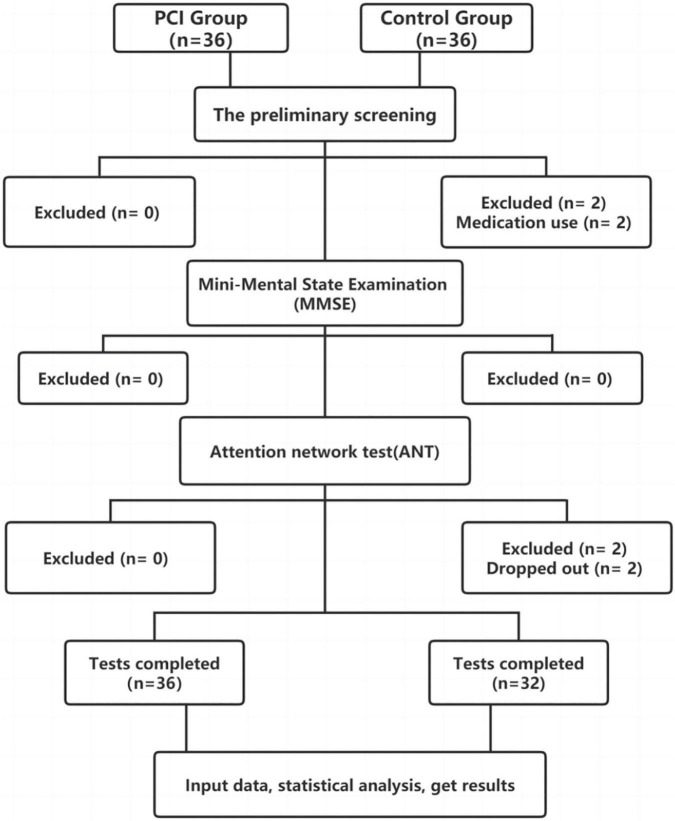
Chart illustrating the participant flow and the reasons for exclusion/withdrawal in the posterior circulation ischemia (PCI) and control groups.

Comparison of the two sets of sample means: The sample size was estimated using the NCSS’s PASS 15.0 sample size estimation software. The primary outcome variables were continuous. According to the results of the Alerting RT pre-experiment of 5 cases in the two groups, the mean and standard deviation of PCI Group and Control Group were estimated, respectively, 29.2 ± 10.99 and 39.2 ± 12.83. The test level α = 0.05 and test efficiency 1-β = 0.8 were determined, and the sample size of the two groups was 1:1 (n1 = n2). After entering the above indicators were input into PASS 15.0 software, the estimated sample sizes of PCI Group and Control Group were both 32 cases. Based on the actual 10% dropout estimate, the sample sizes of the two groups were 36 cases each. The PCI group did not fall off, so the sample size was 36 cases and the control group was 4 cases. Thus, the sample size is 32 cases (Figure 2).

### Subjects

All participants were recruited from the Second Affiliated Hospital of Anhui University of Chinese Medicine and the First Affiliated Hospital of Anhui Medical University. From August 2020 to April 2021, there were 36 inpatients and outpatients with PCI who were assigned to the PCI group, while 32 participants without PCI were included in the control group.

The following diagnostic criteria for PCI were applied, according to multidisciplinary treatment of PCI (Misra et al., 2004): (1) aged 30–75 years old; (2) main symptoms included dizziness or vertigo, accompanied by vomiting, headache, numbness of the head and face or limbs, diplopia, nystagmus, and weakness of the limbs; (3) symptoms may be accompanied by eye movement disorders, visual field defects, hemiplegia, paresthesia, gait changes, ataxia, dysrhythmia, or dysphagia; Horner syndrome; or frequent falling; (4) transcranial Doppler ultrasonography suggesting abnormal hemodynamics in the vertebral basilar artery system; color Doppler ultrasonography suggesting atherosclerotic plaques in the posterior circulation, lumen stenosis, or occlusion; or cranial magnetic resonance imaging (MRI) or computed tomography (CT) examination suggesting brainstem or cerebellar infarction;

The inclusion criteria were: (1) satisfaction of the above diagnostic criteria; (2) a Mini-Mental State Examination (MMSE) score ≥24 points; (3) no obvious audiovisual impairment; (4) right-handed. The inclusion of participants was not limited based on gender.

The exclusion criteria were: (1) vertigo not caused by PCI (such as benign paroxysmal positional vertigo; intracranial space occupation; ocular, otogenic, or drug-induced vertigo; vestibular neuronitis; or Meniere’s disease; (2) MRI or CT examination observed intracranial mass lesions, thalamic infarction or hemorrhage; (3) cognitive dysfunction or mental illness; (4) severe cardiovascular disease, cerebrovascular disease, or abnormal liver and kidney function; (5) current or recent (within 6 months) drug or alcohol abuse or use of medications that may cause memory impairment; and (6) seriously impaired audiovisual functions.

### Neuropsychological background tests and computer program

The MMSE was administered to each participant to assess global cognitive functions. E-prime (Version 2.5, Psychology Software Tools, Pittsburgh, PA, USA) was used to compile the experiment program. The experiment was conducted on a desktop computer.

### Attention Network Test

**FIGURE 3:**
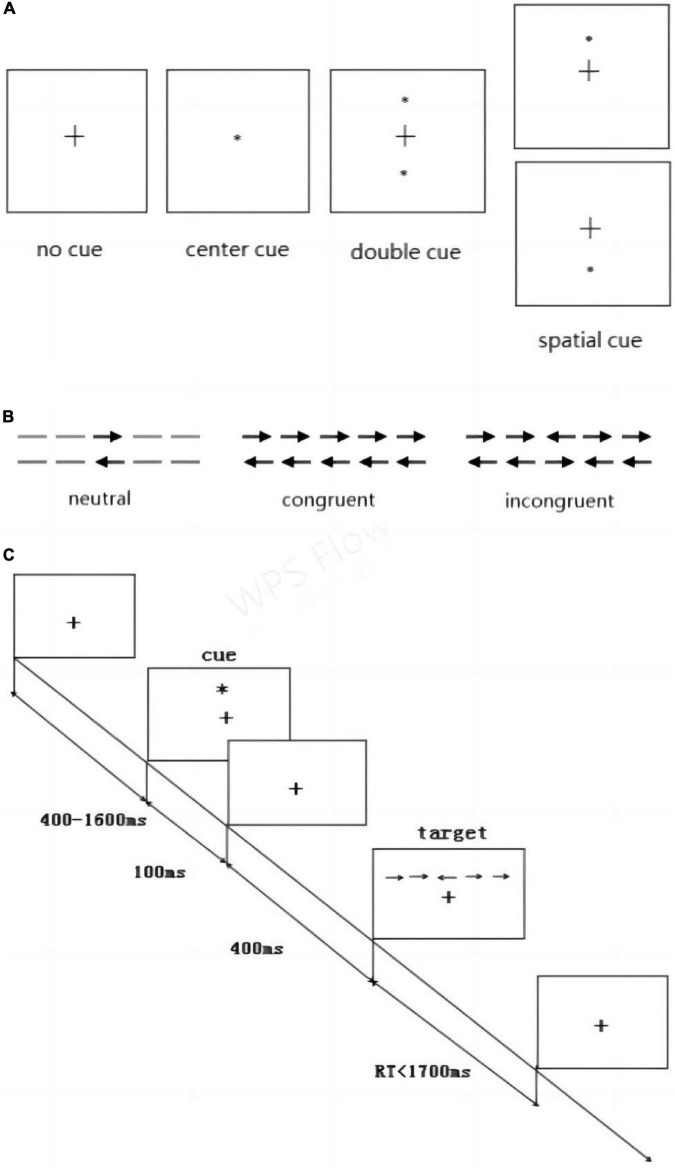
Attention Network Test. **(A)** Four cue stimulus conditions and **(B)** three target stimulus conditions were used in the experiment. **(C)** Example of the procedure.

The ANT designed by Fan et al. (2003) was applied to test the efficiency of the alerting, orienting, and executive control attention networks simultaneously. The participant was placed 60 cm from a computer screen during the test. The gaze remained in the center of the screen, and the participant’s left and right index fingers were placed on the left and right response keys of the keyboard. Three kinds of experimental stimuli were proposed: the fixation point (+), the cue stimulus (*), and the target stimulus (arrow). In the first step, fixation point was displayed at the center of the screen for 400–1600 ms. This indicated the beginning of the experiment. In the second step, cue stimulus was presented for 100 ms in one of four permutations relative to fixation point: no cue, central cue, double cue, and spatial cue (above or below) (Figure 3A). These positions were independent of the location of target stimulus presented next.

In the third step, the fixation point was presented again for 400 ms. Then, in forth step, the participant was presented the target stimulus, which was three horizontal rows of arrows presented in one of three conditions: neutral, congruent, or incongruent (Figure 3B). The participant was instructed to respond as soon as possible to use the keyboard to indicate the direction of the middle arrow. If the middle arrow pointed to the left, the “→” key should be pressed. If the arrow pointed to the right, the “→” key should be pressed. In fifth step, the fixation point was displayed again at the center of the screen.

The experiment consisted of 312 trials, and each cue condition occurred 78 times throughout the experiment, whereas each target condition occurred 104 times, in varying combinations. The computer automatically recorded the average response time (RT) and average accuracy of each participant (Figure 3C) outlines the overall test procedure.

### Calculation of attention network efficiencies

According to the ANT design principle, the efficiencies of alerting, orienting, and executive control can be calculated by subtracting RT under different conditions:

**Alerting RT (ms)** = RT (no cue) -RT (double cue); the greater the difference, the higher the alerting efficiency.

**Orienting RT (ms)** = RT (central cue) -RT (spatial cue); the greater the difference, the higher the orienting efficiency.

**Executive Control RT (ms)** = RT (incongruent target)-RT (congruent target); the greater the difference, the lower the executive control efficiency.

The overall mean RT was calculated as the mean of the three types of RT. Accuracy was calculated from the proportion of correct answers.

### Ethical considerations

All participants had the ability to understand the study procedures. After explaining the purpose of the study, all participants provided their written informed consent. This study was approved by the Ethics Committee of the Second Affiliated Hospital of Anhui University of Chinese Medicine. The work was carried out in accordance with The Code of Ethics of the World Medical Association (Declaration of Helsinki) for experiments involving humans (Shrestha and Dunn, 2020).

### Statistical analysis

SPSS 23.0 (IBM, Armonk, NY, USA) was used for all statistical analyses. SPSS 23.0 was used for normality test. Continuous variables conforming to the normal distribution were described as mean ± standard deviation (SD). Age, years of education, MMSE, Alerting RT, Orienting RT, Executive control RT, Overall mean RT and Accuracy (%) were all consistent with normal distribution. Two independent sample *t*-tests (variance homogeneity) or two independent samples corrected *T*-test (variance heterogeneity) were used to compare the mean between the two groups, and the chi-square test was used to compare the sex composition and risk factors between the two groups. We adopted the correction for multiple comparison that *P* < 0.01 was statistically significant (Bonferroni correction).

## Results

### Clinical data

Participant characteristics of the two groups are shown in Table 1. There were no significant differences between the two groups in sex, age, education level, risk factors, or MMSE scores.

**TABLE 1:** Participant characteristics.

Characteristic	PCI group	Control group	*P*-value
*n*	36	32	
Age in years; Mean ± SD (range)	50.45 ± 10.77 (30–75)	48.2 ± 9.3 (30–75)	0.355**
Sex; Female/male	16/20	18/14	0.466^#^
Years of education; Mean ± SD (range)	8.6 ± 3.2 (0–15)	8.2 ± 2.9 (0–16)	0.627**
MMSE score; Mean ± SD	28.2 ± 1.3	27.3 ± 1.3	0.082**
High-risk factors: Hypertension/diabetes/hyperlipemia	15/8/5	13/7/6	0.893^#^

MMSE, mini-mental state examination; PCI, posterior circulation ischemia; SD, standard deviation. Age, years of education and MMSE were all consistent with normal distribution. **Two independent sample *t*-tests, ^#^Chi-square test. No significant differences were found between the PCI group and the control group.

### ANT index between the PCI group and the control group

**FIGURE 4:**
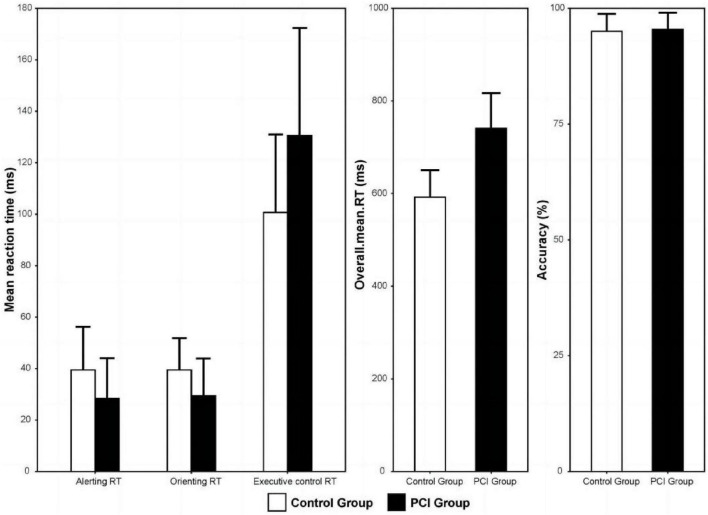
Mean response time of the PCI and control groups for the three kinds of attention network. Error bars represent standard deviation. PCI, posterior circulation ischemia; RT, response time.

Alerting RT and orienting RT in the PCI group were significantly shorter than in the control group (all *P* < 0.01), while executive control RT and overall mean RT in the PCI group were significantly longer than those in the control group (all *P* < 0.01). There was no significant difference in the average accuracy between the two groups (*P* > 0.01; Table 2 and Figure 4).

**TABLE 2:** Comparison of attention network function between controls and patients with posterior circulation ischemia.

Items	PCI group	Control group	*P*-value	Cohen’s d
Alerting RT	28.36 ± 15.62	39.41 ± 16.85	0.007	0.68
Orienting RT	29.42 ± 14.52	39.44 ± 12.37	0.003	0.743
Executive control RT	130.5 ± 41.81	100.66 ± 30.3	0.001	0.817
Overall mean RT	740.33 ± 76.60	592.25 ± 58.25	<0.001	2.176
Accuracy (%)	95.44 ± 3.64	95.03 ± 3.73	0.154	0.111

RTs are reported in milliseconds. All data were normally distributed. All data are reported as mean ± SD. RT, response time; PCI, posterior circulation ischemia. All items showed a significant difference between the two groups (*P* < 0.01), except for accuracy (*P* > 0.01).

## Discussion

In this study, we used ANT to evaluate the difference in attention network function between patients with and without PCI and to investigate which attention network, if any, was affected by PCI. The results showed that ANT can be performed in patients with PCI to determine the total impairment of the alerting, orienting, and executive control attention networks, as well as the overall mean RT. We found deficits in all three networks in patients with PCI. However, we found no significant differences in accuracy between the two groups.

The alerting network refers to the ability to acquire and maintain a highly sensitive state in response to stimuli quickly. Neuroimaging studies have shown that alarm signals are related to the thalamus, the reticular ascending structure of the brainstem, and the frontal and parietal lobes. Neurochemical studies have also found that alertness is linked to the norepinephrine system in the brain. Studies have confirmed that neurovascular unit components are impaired during ischemia and chronic hypoperfusion (Zlokovic, 2008, 2011). During these pathological processes, permeability and selective loss of the blood-brain barrier (Forster et al., 2016), where norepinephrine passes through the blood-brain barrier. Therefore, loss of norepinephrine in the peripheral and central nervous system may result in an impaired alerting network in patients with PCI.

The orienting network refers to the sensory information from the processes involved in selecting the effective stimulus. The parietal lobe, temporoparietal junction, and the frontal vision area, including the cholinergic system, play a key role in this function. A previous study confirmed that rats suffering from chronic cerebral hypoperfusion have different levels of cortical injuries in the brain (Kim et al., 2009). This could be due to damage to the frontal and parietal regions of the cortex, which reduces the efficiency of the orienting network in PCI patients.

Executive control function is the result of the interaction of different brain regions, where the prefrontal cortex is considered a key neural structure to accomplish this function. The dopaminergic system also plays an important role. Executive control of attention has been associated with the midline frontal areas (anterior cingulate cortex, ACC) and lateral prefrontal cortex, which are target areas of the ventral tegmental dopamine system (MacDonald et al., 2000). Studies have shown that PCI can lead to neuronal damage mainly in the hippocampal cornu ammonis, cerebral cortex, thalamus, striatum, and cerebellum (Wang et al., 2019). These block the midbrain-cortex pathway of the dopaminergic system, and deficiencies of dopamine D1 receptors in the dorsolateral prefrontal cortex can impair motor function, prolong response time, and thus reduce the efficiency of the executive network system.

Previous studies have shown that long-term cerebral insufficiency may impair metabolic activities and lead to cognitive dysfunction (Zhao et al., 2013; Lee et al., 2017). Studies have shown that occipital lobe cerebral hypoperfusion is associated with decreased attention and executive processing in Type 2 diabetes mellitus (T2DM) patients (Wang Y. et al., 2021).

Besides, hypoxia has reduced CBF in several brain regions, including the right temporal and bilateral occipital lobes, the anterior and posterior lobes of the cerebellum, the culmen and declive, and the inferior semilunar lobule of the cerebellum, which can lead to cognitive dysfunction (Liu et al., 2021). Similarly, primary open-angle glaucoma (POAG) has been suggested to be a neurodegenerative disease associated with altered cerebral vascular hemodynamics and widespread disruption of neuronal activity within the visual, working memory, attention and executive networks (Wang Q. et al., 2021). PCI is an important risk factor for cognitive impairment and dementia, and its effect on cognitive function is a complex multi-channel process. Improvement of posterior circulation or vertebral artery perfusion improves cognitive function (Rasmussen et al., 2000; Moftakhar et al., 2005). There are three main areas associated with cognitive deficits during posterior circulatory hypoperfusion and stroke. The first is the hippocampus, located in the limbic system, which is an important area for consciousness and memory. The second is the thalamus. Clinical observations have shown that the thalamus is involved in various cognitive functions and psychological activities, including memory, language (language impairment), perception, and emotion. The third major area is the cerebellum. Cerebellar lesions may impair visuospatial abilities and have different characteristics depending on the side where the lesion is present (Molinari et al., 2004).

In addition, patients with transient ischemic attack (TIA) often have cognitive impairment at the time of diagnosis (Moftakhar et al., 2005). Cognitive impairment may be a transient manifestation that may persist until ischemia related symptoms disappear or may persist (Pendlebury and Rothwell, 2009; Pendlebury et al., 2011). Transient ischemic attack (TIA), an important risk factor for stroke (Lv et al., 2021), carry a 10% risk of stroke within 90 days and are therefore considered a serious condition meriting urgent attention. Cognitive deficits including memory, language, perception, emotion, or visual agnosia tend to be underdiagnosed. Therefore, it is important to evaluate the posterior circulation, since transient neurological attacks due to transient attacks precede posterior circulation strokes in patients with vascular risk factors (Paul et al., 2013). Optimal management of modifiable risk factors is further investigated. Routine cognitive assessment in TIA work-up is recommended in order to lessen cognitive dysfunction in these patients with TIA. Early assessment of cognitive function in patients with PCI is particularly important, and ANT is an efficient tool for this assessment. Therefore, our findings provide a direction for the treatment of patients with posterior circulation ischemia, and can play an early prevention role.

## Limitations

This study has some limitations. First of all, this is a small sample size study, which may result in less convincing enough. In the future, we will expand the sample and further study to explore the cognitive mechanism of attention network impairment in patients with posterior circulation ischemia. Second, according to the duration of posterior circulation ischemia, patients with posterior circulation ischemia may show different degrees of cognitive impairment. In the future, we can group patients according to the duration of posterior circulation ischemia to explore whether the cognitive function of patients with posterior circulation ischemia shows progressive impairment. Third, no fMRI data were collected in this study. In the future, we will further collect fMRI data from patients with posterior circulation ischemia, and the correlation between FMRI and attention network function can be studied. Fourth, there was no neurological patient group as a control group. We will expand the sample size in future studies and add the neurological patients groups. Fifth, we only used MMSE as a cognitive test tool for patients, MoCA scale is more suitable for early detection of cognitive impairment than MMSE scale, and we will add the MoCA scale to future cognitive function assessments.

## Conclusion

To our knowledge, this is the first study to use ANT to examine the functioning of the three attention networks in PCI patients. Our results hint an impairment of the alerting, orienting, and executive control attention network and overall prolonged RTs time in PCI patients. Further studies are needed to determine whether attention deficits can be reversed after improved PCI treatment.

## Data availability statement

The raw data supporting the conclusions of this article will be made available by the authors, without undue reservation.

## Ethics statement

The studies involving human participants were reviewed and approved by Ethics Committee of the Second Affiliated Hospital of Anhui University of Chinese Medicine. The patients/participants provided their written informed consent to participate in this study.

## Author contributions

NL participated in the conception and design of the study, data collection, and manuscript writing. CL participated in the data collection and made diagrams. GL and KW proposed ideas for this research. XX revised the manuscript and made diagrams. GL analyzed and interpreted the data and visualized the results. WZ and JF reviewed and made significant revisions to the manuscript. All authors were involved in revising the manuscript and agreed to the published version of the manuscript.
